# Deletion of *Fbxw7* in oocytes causes follicle loss and premature ovarian insufficiency in mice

**DOI:** 10.1111/jcmm.18487

**Published:** 2024-06-21

**Authors:** Huihui Zhao, Hanbin Zhang, Yuxia Zhou, Ling Shuai, Zhenguo Chen, Liping Wang

**Affiliations:** ^1^ Department of Cell Biology, School of Basic Medical Sciences Southern Medical University Guangzhou Guangdong P.R. China; ^2^ Guangdong Provincial People's Hospital Southern Medical University Guangzhou Guangdong P.R. China; ^3^ Department of Obstetrics and Gynecology, Key Laboratory for Major Obstetric Diseases of Guangdong Province, Key Laboratory of Reproduction and Genetics of Guangdong Higher Education Institutes The Third Affiliated Hospital of Guangzhou Medical University Guangzhou Guangdong P. R. China; ^4^ Department of Obstetrics and Gynecology, Guangdong Second Provincial General Hospital Guangzhou Guangdong P.R. China; ^5^ Department of Reproductive medicine, Shenzhen Second People's Hospital Shenzhen Guangdong P.R. China

**Keywords:** *Fbxw7*, follicle loss, ovarian dysfunction, premature ovarian insufficiency

## Abstract

Premature ovarian insufficiency (POI) is one of the important causes of female infertility. Yet the aetiology for POI is still elusive. FBXW7 (F‐box with 7 tandem WD) is one of the important components of the Skp1‐Cullin1‐F‐box (SCF) E3 ubiquitin ligase. FBXW7 can regulate cell growth, survival and pluripotency through mediating ubiquitylation and degradation of target proteins via triggering the ubiquitin‐proteasome system, and is associated with tumorigenesis, haematopoiesis and testis development. However, evidence establishing the function of FBXW7 in ovary is still lacking. Here, we showed that FBXW7 protein level was significantly decreased in the ovaries of the cisplatin‐induced POI mouse model. We further showed that mice with oocyte‐specific deletion of *Fbxw7* demonstrated POI, characterized with folliculogenic defects, early depletion of follicle reserve, disordered hormonal secretion, ovarian dysfunction and female infertility. Impaired oocyte‐GCs communication, manifested as down‐regulation of connexin 37, may contribute to follicular development failure in the *Fbxw7*‐mutant mice. Furthermore, single‐cell RNA sequencing and in situ hybridization results indicated an accumulation of *Clu* and *Ccl2* transcripts, which may alter follicle microenvironment deleterious to oocyte development and accelerate POI. Our results establish the important role of *Fbxw7* in folliculogenesis and ovarian function, and might provide valuable information for understanding POI and female infertility.

## 1 INTRODUCTION

The reproductive lifespan of women is from adolescence to menopause, in which the follicle stockpile would exhaust continuously throughout every stages.^1^ Premature ovarian insufficiency (POI) is characterized by amenorrhea for at least 4 months and an elevated follicle‐stimulating hormone (FSH) level (>25 IU/L) on two occasions at least 1 month apart before the age of 40.^2^ POI is one of the biggest challenges for female infertility and assisted reproductive technology.^3^ The causes for POI are various, which includes iatrogenic or environmental factors, viral infections, metabolic and autoimmune diseases, and genetic predisposition.^4^ However, the molecular pathway underlying normal folliculogenesis and the etiopathogenesis of POI are still elusive. Genetic alterations have been recognized for long as a common cause of POI.^5^ The candidate genes potentially causing POI is increasing, while identifying exact causative genes has been challenging, with many discoveries not yet confirmed.^6^, ^7^ Elucidating the gene function is very significance for POI diagnosis and even therapy.

F‐box and WD40 repeat‐containing 7 (FBXW7) is a member of the F‐box protein family and serves as the substrate recognition component of the skp1‐cullin1‐F‐box protein complex (SCF), an E3 ubiquitin ligase.^8^ FBXW7 is an important tumour suppressor that regulates ovarian cancer cell proliferation and migration activity by modulating the ubiquitin‐proteasome system (UPS).^9^, ^10^ In male germ cells, FBXW7 regulates the transition of spermatogonial stem cells from self‐renewal to differentiation through its interaction with MYC.^11^ However, the role of FBXW7 in folliculogenesis and ovarian function has not been characterized.

Folliculogenesis, which encompasses the growth and maturation of follicle units, involves a comprehensive series of cellular processes, including the migration of primordial germ cells, germ cell proliferation, germ cell differentiation and meiosis.^12^, ^13^ This highly orchestrated process is regulated by complex interactions among endocrine factors, follicular autocrine/paracrine signals and adjacent follicular cells.^14^ Early follicles reside in a relatively avascular environment, making them susceptible to microenvironmental influences.^15^ Within the follicle, granulosa cells (GCs), which directly contact the oocyte, engage in direct substance exchange via gap junctions.^16^ GCs mediate oocyte development by secreting EDF‐like factors.^17^, ^18^ However, the mechanisms by which oocyte regulates GCs function remain incompletely understood.

In this study, we found that FBXW7 protein level decreased significantly in the ovaries of cisplatin‐induced POI mouse model. FBXW7 exhibited strong expression in follicles at all developmental stages. The mice with oocyte‐specific deletion of *Fbxw7* gene demonstrated ovarian atrophy, follicle loss and ultimately female infertility. Additionally, we identified two oocyte‐expressing proteins as potential downstream proteins of FBXW7. In summary, our work demonstrated the crucial role of *Fbxw7* in oocyte development and ovarian function, and suggested that *Fbxw7* may regulate the connections between oocytes and GCs by affecting gap junction proteins.

## 2 MATERIALS AND METHODS

### 2.1 POI model

Wild type (WT) female C57BL/6J mice of 1‐month‐old were purchased from the Southern Medical University Animal Center (Guangzhou, China). All mice were housed in a constant light‐to‐dark ratio of 12:12 h under specific pathogen‐free conditions, and were acclimated for **7** days before the experiment. Experimental POI model was generated as described in our previous work,^19^ briefly, by intraperitoneal injections of cisplatin (CDDP, 2.5 mg/kg/d in saline, Sigma‐Aldrich, Shanghai, China) for seven consecutive days, while the control group was injected with saline of same volume. All animal experiments were approved by the Southern Medical University Committee on the Use and Care of Animals and were performed in accordance with the Committee's guidelines and regulations.

### 2.2 Mutant mice, husbandry and genotyping

Growth differentiation factor 9 (*Gdf9*)‐Cre mice were obtained from the Jackson Laboratory (Stock number: 011062). *Fbxw7*‐loxP mice were used in our previous study,^20^ and followed the same mating strategy to obtain female mice homozygous for *Fbxw7*‐loxP and heterozygous for *Gdf9*‐Cre (*Gdf9*‐Cre^+^, *Fbxw7* ^loxP/loxP^). These females carried an oocyte‐specific deletion of *Fbxw7*, and were designated *Fbxw7* OcKO mice and the females from the same litter homozygous for *Fbxw7*‐loxP but without *Gdf9‐*Cre (*Gdf9*‐Cre^−^, *Fbxw7* ^loxP/loxP^) were used as control mice. DNA was isolated from tail biopsies and used to genotype the mice by PCR with the primer sequences below:

*Gdf9‐*forward: TCTGATGAAGTCAGGAAGAACC.

*Gdf9*‐reverse: GAGATGTCCTTCACTCTGATTC.

*Fbxw7*‐Forward: ATTGATACAAACTGGAGACGAGG.

*Fbxw7*‐reverse: ATAGTAATCCTCCTGCCTTGGC.

### 2.3 Tissue collection and histology

Ovaries were fixed in 4% paraformaldehyde solution for 12 h and then dehydrated, embedded in paraffin wax. Five micron thickness of sections was prepared for haematoxylin and eosin (H&E) staining. The number of follicles of each ovary was determined by counting the follicles containing oocytes with a visible nucleus,^21^ and every fifth section was assessed. Primordial, primary, secondary and antral follicles were classified as described by Pedersen and Peters.^22^

For immunofluorescence (IF), sections were incubated with primary and secondary antibodies below: rabbit anti‐ FBXW7 primary antibody (1:100, A5872, ABclonal technology, Wuhan, China), rabbit anti‐connexin 37 primary antibody (1:100, A2529, ABclonal technology, Wuhan, China), rabbit anti‐Ki‐67 primary antibody (1:300, A9129, ABclonal, Wuhan, China), rabbit anti‐connexin 43 primary antibody (1:100, YT1046, Immunoway, Jiangsu, China); Alexa‐Fluor‐488‐ or Alexa‐Fluor‐594‐labelled secondary antibodies (Jackson Immunoresearch, West Grove, PA, USA). The sections were finally counterstained with DAPI and immunofluorescent images were acquired using a FluoView FV1000 confocal microscope (Olympus, Tokyo, Japan).

### 2.4 Immunoblotting

After euthanasia, ovaries were immediately removed and lysed and boiled in 2% SDS lysis buffer containing protease inhibitor. The protein extracts were then subjected to 6%–12% SDS‐PAGE and electrotransferred to nitrocellulose membranes (GE Healthcare Life Sciences, Beijing, China). The membranes were blocked with 5% non‐fat dry milk for 1 h at room temperature, washed and incubated with the indicated primary antibody at 4°C overnight. The membranes were further washed, incubated with Peroxidase‐AffiniPure Goat Anti‐Rabbit or Mouse IgG (H + L) (Jackson ImmunoResearch, West Grove, PA, USA) for 1 h at room temperature, washed again and finally visualized with an enhanced chemiluminescence kit (PerkinElmer, Waltham, MA, USA). α‐Tubulin served as an internal control (mouse anti‐α‐Tubulin primary antibody, 1:5000, T6074, Sigma‐Aldrich). The quantitative analysis of protein expression was carried out with ImageJ software 1.8.0 (NIH, Bethesda, Maryland, USA).

### 2.5 Quantitative real‐time PCR

Total ovarian and cell line RNA was purified using Oligotex mRNA Mini Kit (QIAGEN, 70022, Shenzhen, China), processed to cDNA and amplified target genes using a PrimeScript™ RT reagent Kit (Takara, RR037Q, Beijing, China), then quantified them by StepOne Plus Real‐Time 210 PCR System (Applied Biosystems, Waltham, MA, USA). Glyceraldehyde 3‐phosphate dehydrogenase (*Gapdh*) was used as the endogenous control transcript. The primer sequences are summarized below:

*Gapdh‐*forward: TGGAAGGACTCATGACCACA.

*Gapdh‐*reverse: TTCAGGTCAGGGATGACCTT.

*Fbxw7*‐forward: ACTGGGCTTGTACCATGTTCA.

*Fbxw7*‐reverse: TGAGGTCCCCAAAAGTTGTTG.

*Ccl2*‐forward: GGCCAAGGAGATCTGTGCTGAC.

*Ccl2*‐reverse: TGGAGTGAGTGTTCAAGTCTTCGG.

*Clu*‐forward: CCAATCAGGGAAGTAAGTACGTC.

*Clu*‐reverse: CTTGCGCTCTTCGTTTGTTTT.

### 2.6 Cell apoptosis by TdT‐mediated dUTP Nick‐End Labeling (TUNEL) analysis

Cell apoptosis in the follicles was evaluated in sections using a TUNEL assay for the situ visualization of DNA fragmentation with the commercial DeadEnd™ Fluorometric TUNEL System (G3250, Promega, Madison, WI, USA). Images were obtained by a FluoView FV1000 confocal microscope. Every 25th section in each ovary was analysed. Number of apoptotic granulosa cells indicates extent of each follicle injury in each section.

### 2.7 Serum hormone measurement by enzyme‐linked immunosorbent assay (ELISA)

Mice were anaesthetised to collect their blood via cardiocentesis. The blood samples were centrifuged at 3000× *g* for 10 min and the serum collected. The plasma levels of anti‐Mullerian hormone (AMH), inhibin B (INHB), estradiol (E2), follicle stimulating hormone (FSH), luteinizing hormone (LH) and progesterone (P) were measured with commercial ELISA kits (Elabscience Biotechnology, Wuhan, China).

### 2.8 Fertility evaluation

Female mice were mated with adult WT males with proven fertility at a ratio of 2:1 for 6 months. Pregnant females were then separated and litter sizes were recorded on delivery.

### 2.9 In situ hybridization

In situ hybridization was conducted on paraffin‐embedded specimens (4 μm thickness). Paraffin sections were deparaffinized in xylene and rehydrated in graded alcohols and distilled water. After treating with proteinase K at 37 th for 30 min, sections were rinsed, fixed, and then prehybridized for 2 h. Hybridization was performed with *Ccl2* and *Clu* Digoxygenin (DIG)‐labelled probes designed and synthesized by axl‐bio (Guangzhou, China). Slides were hybridized with DIF‐labelled LNA probes overnight at 37 °C and were then washed and incubated with anti‐DIG‐HRP Fab fragments for 1 h at room temperature. Signals were visualized with the 3,3′‐Diaminobenzidine (DAB) substrate (Maixin Biotech. Co., Ltd., Fuzhou, China). Densitometry analysis of the in situ hybridization images were performed with ImageJ software 1.8.0.^23^

### 2.10 Superovulation and oocytes collection

Oocytes were collected from 1‐month‐old paired females, which were injected intraperitoneally with 5 IU Given Pregnant Mare Serum Gonadotropin (PMSG) (Sigma, St. Louis, MO), and after 48 h with 5 IU human chorionic gonadotrophin (hCG). Unfertilized oocytes at metaphase of the second meiotic division (MII) were collected from oviducts 14 h after hCG injection. The cumulus‐oocyte complexes were disassociated from the oviducts and digested with hyaluronidase, and oocytes were collected in M2 medium (Sigma, St. Louis, MO), then captured with a stereoscope (Olympus, SZX7‐4122RFL‐2).

### 2.11 Single cell RNA‐seq data pre‐processing and quality control

Every 40 oocytes were set as one sample. Oocyte RNA were extracted using the Trizol (ThermoFisher) according to the user manual. Raw data (raw reads) of fastq format were firstly processed through in‐house perl scripts. In this step, clean data (clean reads) were obtained by removing reads containing adapter, reads containing ploy‐N and low‐quality reads from raw data. At the same time, Q20, Q30, GC‐content and sequence duplication level of the clean data were calculated. All the downstream analyses were based on clean data with high quality. The adaptor sequences and low‐quality sequence reads were removed from the data sets. Raw sequences were transformed into clean reads after data processing. These clean reads were then mapped to the reference genome sequence. Only reads with a perfect match or one mismatch were further analysed and annotated based on the reference genome. HISAT2 tools software were used to map to reference genome. Gene function was annotated based on the following databases: NR (NCBI non‐redundant protein sequences database); Nt (NCBI non‐redundant nucleotide sequences); Pfam (The database of Homologous protein family); COG (The database of Clusters of Protein homology); Swiss‐Prot (A manually annotated non‐redundant protein sequence database); KOG (The database of Clusters of protein homology); KEGG(The database of Kyoto Encyclopedia of Genes and Genomes); GO (Gene Ontology database). Gene expression levels were estimated by fragments per kilobase of transcript per million fragments mapped (FPKM). The formula is shown as follow: formula of FPKM. For the samples with biological replicates: different expression analysis of two conditions/groups was performed using the DESeq R packag. The resulting FDR (false discovery rate) were adjusted using the PPDE (posterior probability of being DE).The FDR <0.05 & |log_2_(fold change)| ≥1 was set as the threshold for significantly differential expression. Gene Ontology (GO) enrichment analysis of the differentially expressed genes (DEGs) was implemented by the clusterProfiler R package. Enrichment analysis uses hypergeometric testing to find GO entries that are significantly enriched compared to the entire genome background. GSEA (Gene Set Enrichment Analysis) can also be analysed by clusterProfiler. KEGG is a database resource for understanding high‐level functions and utilities of the biological system, such as the cell, the organism and the ecosystem, from molecular‐level information, especially large‐scale molecular datasets generated by genome sequencing and other high‐throughput experimental technologies (http://www.genome.jp/kegg/). We used KOBAS software to test the statistical enrichment of differential expression genes in KEGG pathways. We used clusterProfiler R packages to find KEGG pathway that are significantly enriched compared to the entire genome background. The sequences of the DEGs was blast (blastx) to the genome of a related species (the protein–protein interaction of which exists in the STRING database: http://string‐db.org/) to get the predicted PPI of these DEGs. Then the PPI of these DEGs were visualized in Cytoscape.

### 2.12 Cell culture and treatments

Human granulosa tumour (KGN) cells were cultured in DMEM/ F12 supplemented with 10% FBS, under 5% CO_2_ and 95% humidity. The *CCL2* and *CLU* plasmids (Genechem, Shanghai, China) were transfected using Lipofectamine 3000 and electroporator in accordance with the manufacturer's instructions.

### 2.13 Data management and statistical analysis

Data production and statistical analysis were performed using GraphPad Prism version 8.0 (GraphPad Software, San Diego, CA). Data are presented as mean ± SEM. Differences between groups were analysed with *t*‐test, and among groups with one‐way ANOVA followed by the post hoc Tukey test. *P* value <0.05 was considered statistically significant.

## 3 RESULTS

### 3.1 FBXW7 is associated with CDDP‐induced POI

**FIGURE 1:**
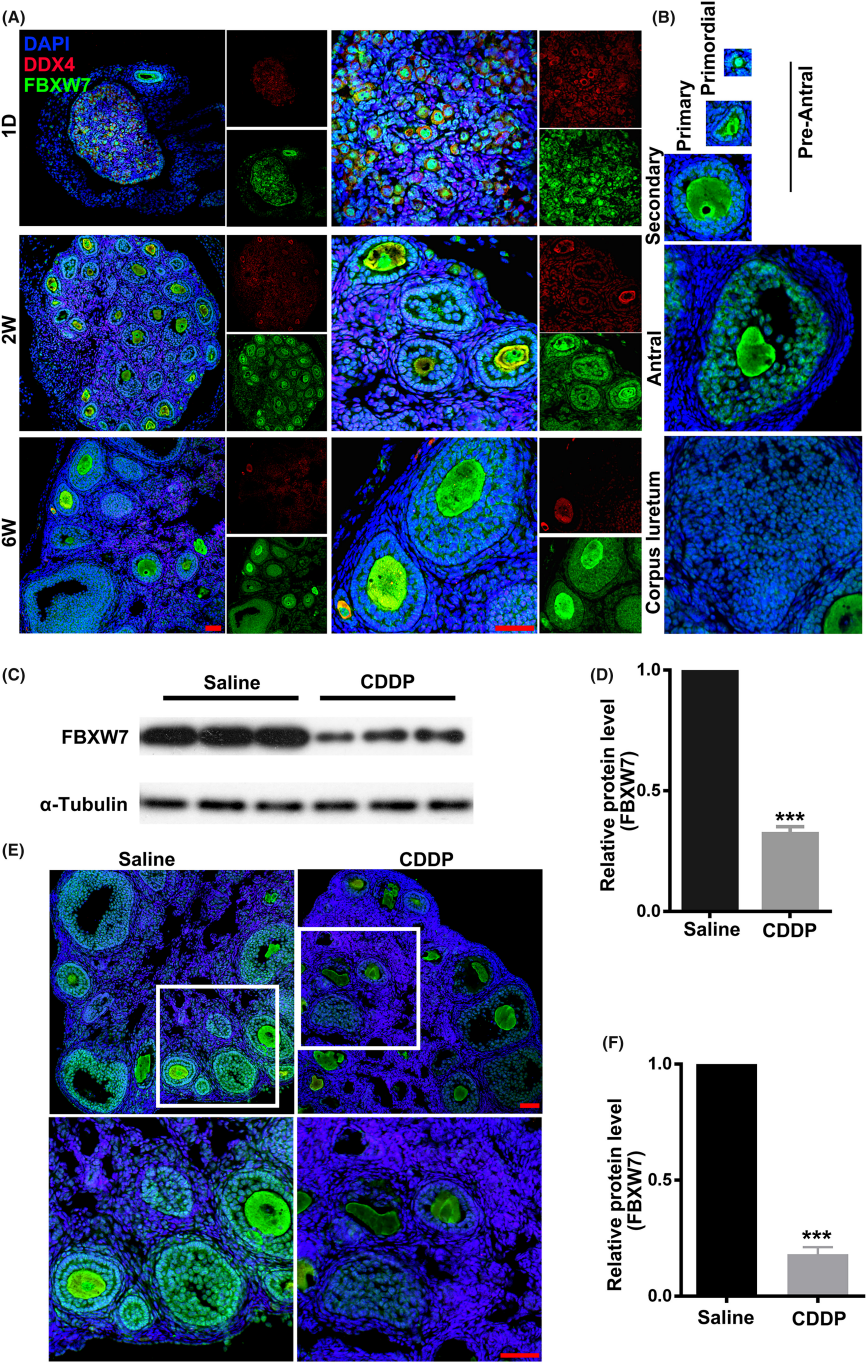
FBXW7 is associated with CDDP‐induced POI. (A) Co‐immunofluorescence of FBXW7 (green) and DDX4 (an oocyte marker, red) in the ovaries from 1‐day‐, 2‐week‐ and 6‐week‐old WT female mice. (B) FBXW7 is expressed in both oocytes and granulosa cells (GCs) of follicles at all developmental stages, including primordial, primary, secondary and antral follicles, and corpus luteum. (C) Western blotting of FBXW7 in the ovaries from CDDP‐induced POI model and saline‐treated control mice. (D) Quantification of the results in C. (E) Immunofluorescent staining of FBXW7 in the ovaries from CDDP‐ and saline‐treated mice. (F) Quantification of the results in E. Data were presented as mean ± SEM. *n* = 3 for each group, scale bar = 50 μm. ***, *p* < 0.001, compared with control.

To reveal the dynamic changes of FBXW7 during ovarian development, we examined the expression of FBXW7 in the ovaries of mice at the ages of 1‐day, 2‐week‐old, and 6‐week‐old, respectively (Figure 1A). FBXW7 was expressed in follicle at all developmental stages, and its expression level kept steady with follicular development. In individual follicles, FBXW7 was distributed in both the cytoplasm and nucleus of oocytes and granulosa cells (GCs), with significantly higher levels in oocytes than in GCs (Figure 1B). However, FBXW7 was not expressed in theca cells. This indicates that FBXW7 may play a role in folliculogenesis and oocyte maturation. To explore the relationship between FBXW7 expression and the ovarian damage caused by the chemotherapy drug CDDP, we established a CDDP‐induced POI model by intraperitoneally injecting CDDP into WT C57B6/J female mice. Western blotting showed a significant reduction in total FBXW7 expression in the ovaries following CDDP treatment (Figure 1C,D). IF staining on tissue sections confirmed a significant downregulation of FBXW7 in follicles (Figure 1E,F). These findings suggest that FBXW7 is downregulated following CDDP‐induced ovarian damage, linking to POI.

### 3.2 Generation of oocyte‐specific *Fbxw7* knockout mice

**FIGURE 2:**
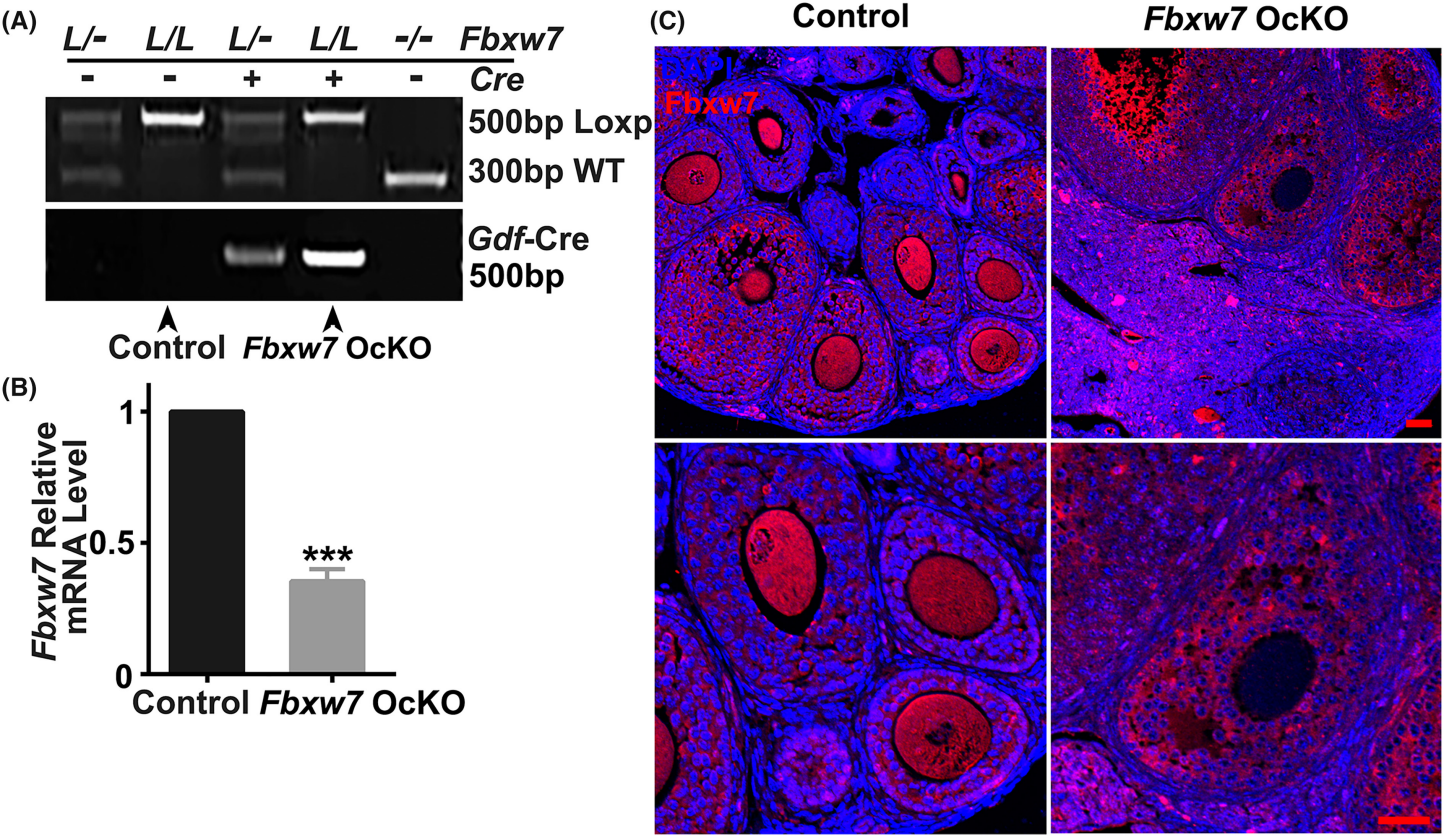
Generation of oocyte‐specific *Fbxw7* knockout mice. (A) Genotyping of the offspring after mating the mice with the *Gdf9*‐Cre and the *Fbxw7*‐loxP allele. The oocyte‐specific *Fbxw7* knockout mice were designated as *Fbxw7* OcKO mice. (B) Assessment of the *Fbxw7* mRNA levels by qPCR in the ovaries of control and *Fbxw7* OcKO mice. mRNA levels were normalized against *Gapdh*. (C) Immunofluorescence of FBXW7 in the ovaries from the paired mice. All mice used were 6‐week‐old. Data were presented as mean ± SEM. *n* = 3 for each group, scale bar = 50 μm. ***, *p* < 0.001, compared with control.

To further investigate the role of FBXW7 in folliculogenesis, we crossed the mice with homozygous floxed *Fbxw7* allele (*Fbxw7* ^loxP/loxP^) with the mice expressing Cre under the control of the *Gdf 9* promoter, which becomes active in oocytes at postnatal Day 3. We referred to the resulting conditional knockout mice as *Fbxw7* OcKO mice. PCR analysis confirmed the genotype of the conditional knockout mice and the litter control mice (Figure 2A). To assess the efficiency of *Fbxw7* knockout, we performed qPCR analyses of *Fbxw7* mRNA levels in the ovaries of 2‐week‐old knockout and control mice (Figure 2B). The results showed a significant decrease in the *Fbxw7* mRNA levels in the *Fbxw7* OcKO ovaries. We further conducted immunofluorescence staining of FBXW7 on ovarian tissue sections from 6‐week‐old paired mice, which revealed the absence of FBXW7 expression in both primordial and secondary oocytes in the *Fbxw7* OcKO ovaries (Figure 2C). These results indicate the successful knockout of *Fbxw7* gene in oocytes of *Fbxw7* OcKO mice.

### 3.3 Loss of *Fbxw7* in oocytes leads to ovarian atrophy, follicle loss, and female infertility

**FIGURE 3:**
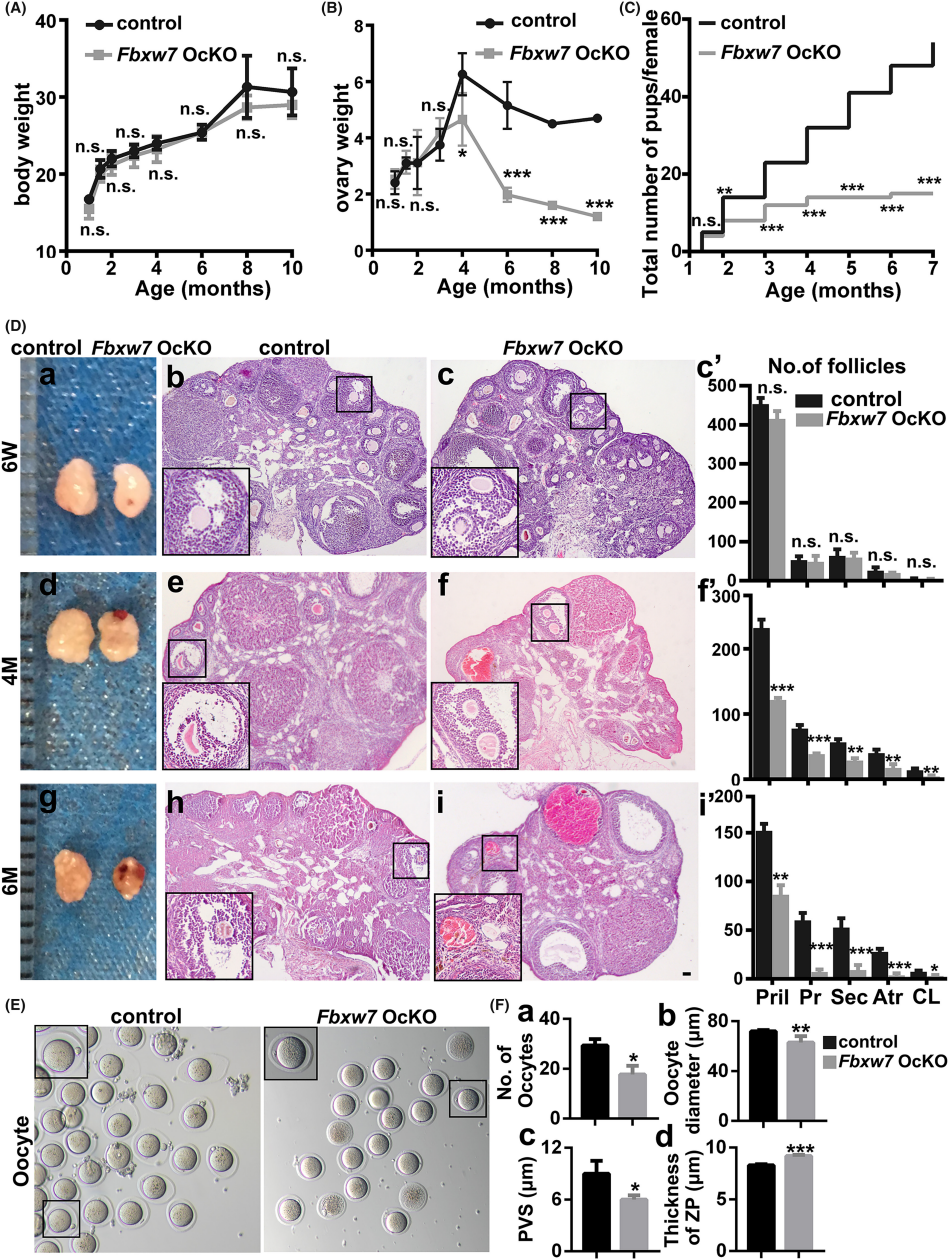
Loss of *Fbxw7* in oocytes leads to ovarian atrophy, follicle loss, and female infertility. (A–C) Age‐dependent changes of body weight (A), ovary weight (B), and cumulative numbers of pups (C) in the control and *Fbxw7* OcKO mice. Note that *Fbxw7* OcKO females were infertile after the age of 6 months old. (D) Ovarian and follicular morphology by H&E staining of 6‐week‐ (a–c), 4‐month‐ (d–f) and 6‐month‐old (g–i) control and *Fbxw7 O*cKO mice, respectively. c’, f’ and i’ show the quantitative and statistical analysis of the numbers of follicles at indicated ages, including primordial (Pril), primary (Pr), secondary (Sec), and antral follicles (Atr) and corpus luteum (CL). (E, F) Morphology, quantity, diameter, periovular space (PVS) and zona pellucida (ZP) thickness of MII‐oocytes in control and *Fbxw7* OcKO mice obtained by superovulation. The inserts present a higher magnification of the area within the black box. Data were presented as mean values ± SEM. *n* = 6–10 for each group, scale bar = 50 μm. n.s., not significant. *, *p* < 0.05; **, *p* < 0.01; ***, *p* < 0.001, compared with control.

We then explored the phenotypes of the *Fbxw7* OcKO mice. There was no significant difference in the body weight between the *Fbxw7* OcKO and control mice (Figure 3A). The ovary weight in the *Fbxw7* OcKO mice significantly decreased since the age of 4 months (*p* < 0.05), only reaching 38%, 44%, and 34% of those in the control group at the age of 6, 8 and 10 months, respectively (Figure 3B). Starting from the age of 2 months, *Fbxw7* OcKO mice exhibited significantly reduced cumulative litter sizes (*p* < 0.01), which continued to decline with increasing age (*p* < 0.001), eventually leading to infertility by 7 months (Figure 3C). To explore whether the infertility in *Fbxw7* OcKO mice resulted from progressive follicle loss, we conducted histological analyses of ovaries (Figure 3D). At the aged of 6 weeks, the ovarian morphology and follicular structure of *Fbxw7* OcKO mice appeared similar to those of control mice, with no significant differences in the number of follicles at different developmental stages. However, at the ages of 4 and 6 months, *Fbxw7* OcKO ovaries exhibited atrophy, noticeable hemorrhagic spots, and a significant reduction in the number of follicles at all developmental stages. Notably, *Fbxw7* OcKO mice had nearly lost all follicles except primordial follicles by 6 months. To investigate the impact of *Fbxw7* deficiency on oocyte development and quality, we analysed the nuclear morphology, oocyte quantity (Figure 3E), diameter, perivitelline space (PVS), and zona pellucida (ZP) thickness (Figure 3F) of MII stage oocytes obtained through superovulation. PVS determines embryo quality, with larger PVS has been shown to enhance human embryo quality^24^; cytoplasmic volume is a critical determinant of oocyte quality, indicating the quantity of organelles and metabolites that influence overall oocyte quality^25^; ZP thickness is directly correlated with oocyte fertilization, with thicker ZP leading to failed fertilization.^26^ Compared to control oocytes, *Fbxw7* OcKO oocytes exhibited significant reduced quantity, decreased oocyte diameter and PVS, while increased ZP thickness and cell death. These results collectively suggested the crucial role of FBXW7 in follicular development, with its loss directly resulting in follicle loss, decreased oocyte quality and quantity, and ultimately female infertility.

### 3.4 Loss of *Fbxw7* in oocytes leads to POI

**FIGURE 4:**
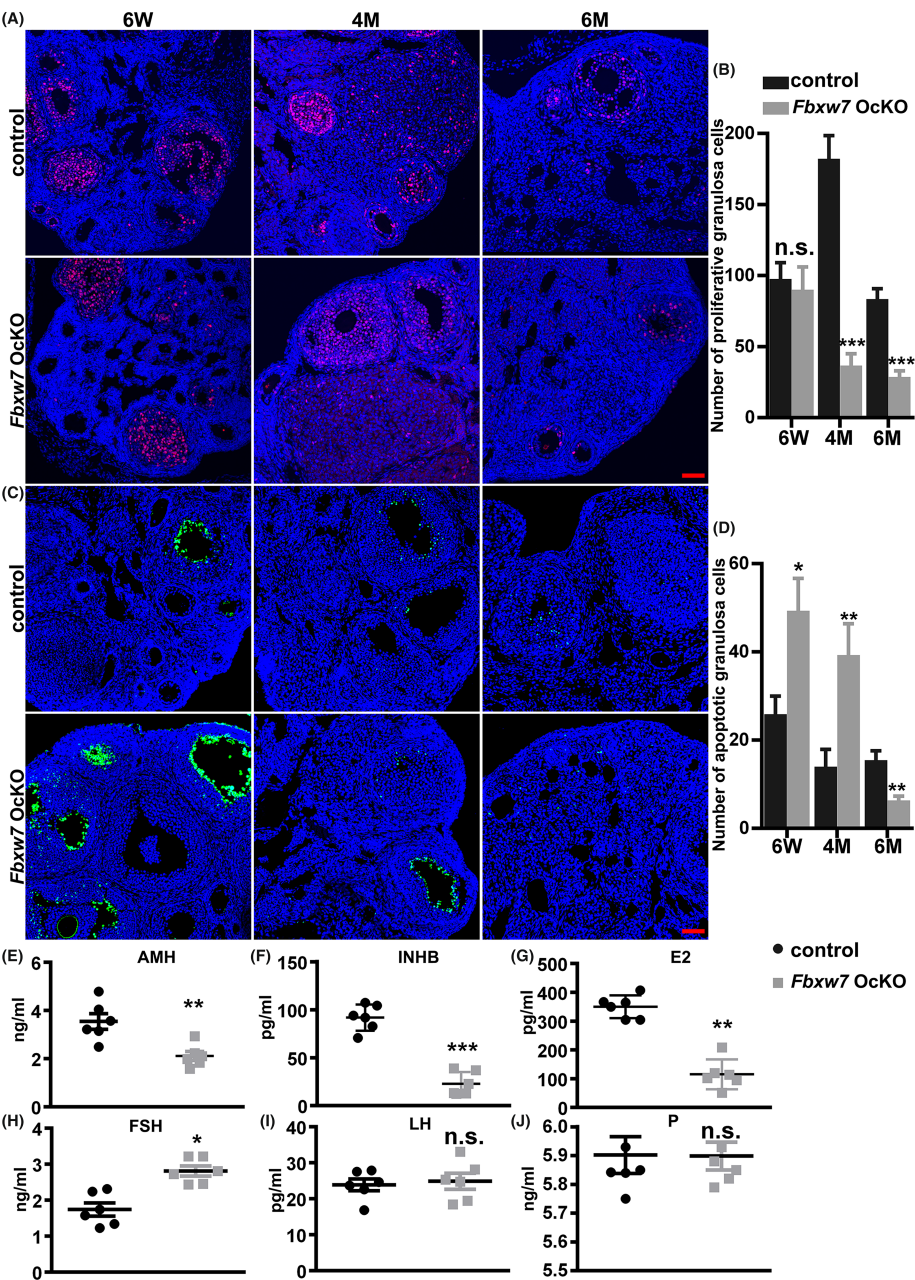
Loss of *Fbxw7* in oocytes leads to POI. (A, B) Cell proliferation assay by immunofluorescent staining of Ki‐67 (red) and quantification. (C, D) Cell apoptosis assay by immunofluorescent TUNEL staining (green) and quantification. (E–J) Assessment of plasma sex hormone (AMH, INHB, E_2_, FSH, LH and P) levels by ELISA assay. Scale bar = 50 μm. Data were presented as mean values ± SEM. *n* = 6 for each group, scale bar = 50 μm. n.s., not significant. *, *p* < 0.05; **, *p* < 0.01, ***, *p* < 0.001, compared with control.

To explore the reasons for the follicle loss observed in *Fbxw7* OcKO mice, we examined cell proliferation and apoptosis within follicles (Figure 4A–D). The results showed increased GC cell apoptosis in the follicles of 6‐week‐old *Fbxw7* OcKO mice, reduced GC proliferation and increased GC apoptosis in the follicles at 4 months, as well as reduced GC proliferation at 6 months. Additionally, the serum levels of AMH, INHB, and E2 were significantly decreased, while the FSH level was abnormally elevated, in the *Fbxw7* OcKO mice, and the LH and P levels remained unchanged (Figure 4E–H). These results indicate that *Fbxw7* OcKO mice exhibit significant signs of POI.

### 3.5 Loss of *Fbxw7* in oocytes impairs oocyte‐GC gap junctions and induces GC apoptosis and ovarian dysfunction

**FIGURE 5:**
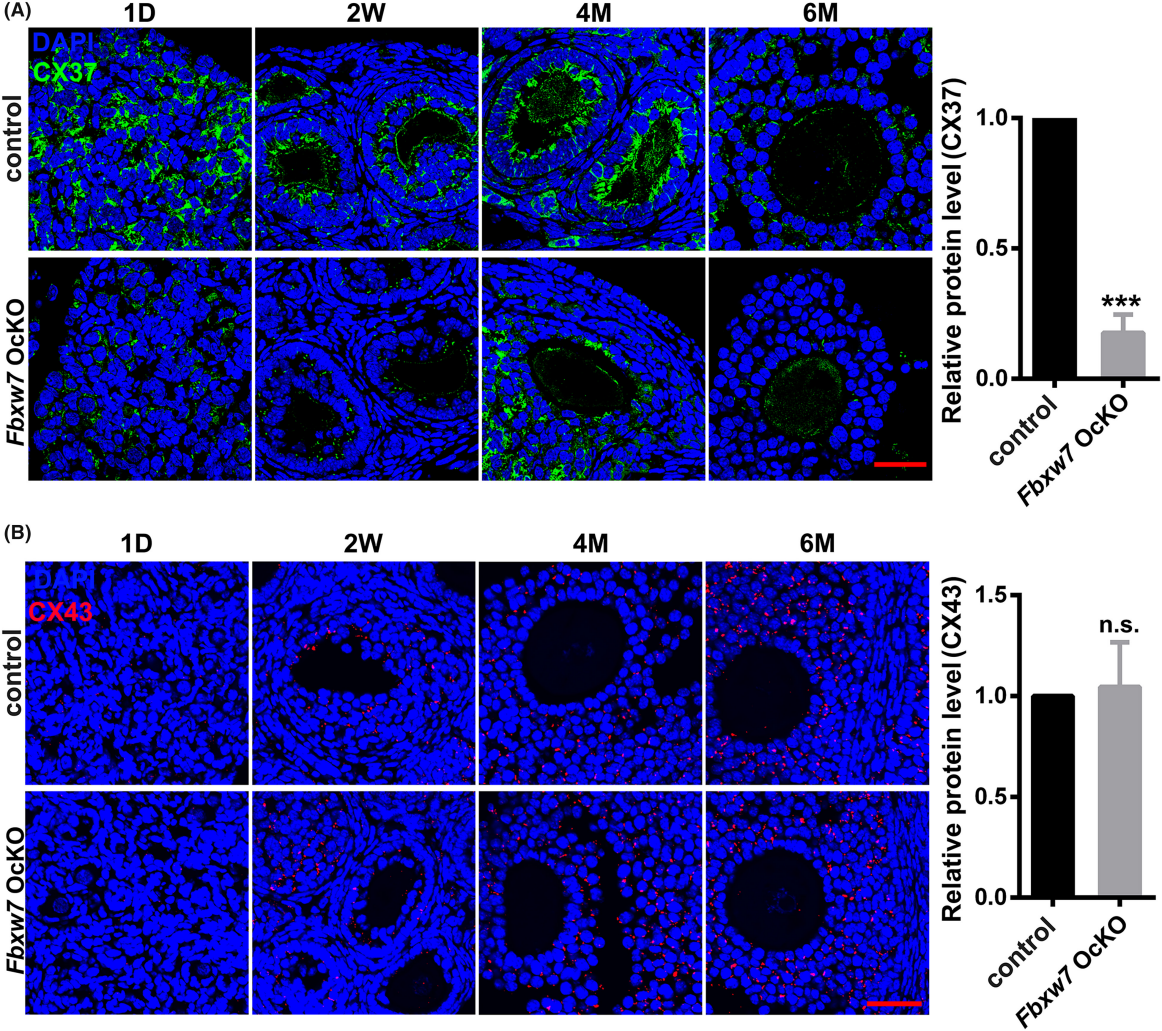
Loss of *Fbxw7* in oocytes impairs oocyte‐GC gap junctions and induces GC apoptosis and ovarian dysfunction. (A) Distribution and expression of Cx37 (a gap junction protein for oocytes‐GCs, green) by immunofluorescence in the ovaries of paired mice at the ages of 1‐day, 2‐week‐, 4‐month‐ and 6‐month‐old. (B) Distribution and expression of Cx43 (a gap junction protein for GCs‐GCs, red) by immunofluorescence. Data were presented as mean values ± SEM. *n* = 5 for each group, scale bar = 50 μm. n.s., not significant. ***, *p* < 0.001, compared with control.

Folliculogenesis relies on the synchronized temporal and spatial coordination of oocyte maturation and GCs proliferation. This process is critically dependent on bidirectional communication established through paracrine signalling and gap junctions between oocytes and GCs. Connexin37 (Cx37) and connexin43 (Cx43) are the principal gap junction proteins involved in the communication between oocytes and GCs, as well as among GCs themselves. To investigate whether the loss of *Fbxw7* in oocytes directly affects GCs function, we performed IF staining of Cx37 and Cx43 in ovarian tissues from 1‐day, 2‐week‐old, 4‐month‐old and 6‐month‐old paired mice (Figure 5A,B). The results revealed a significant reduction in Cx37 expression in the *Fbxw7* OcKO ovaries compared to the controls, while Cx43 expression showed no change. These findings indicate that the absence of *Fbxw7* predominantly disrupts communication between oocytes and GCs.

### 3.6 Deletion of *Fbxw7* causes aberrant elevation of the transcripts of *Ccl2* and *Clu*

**FIGURE 6:**
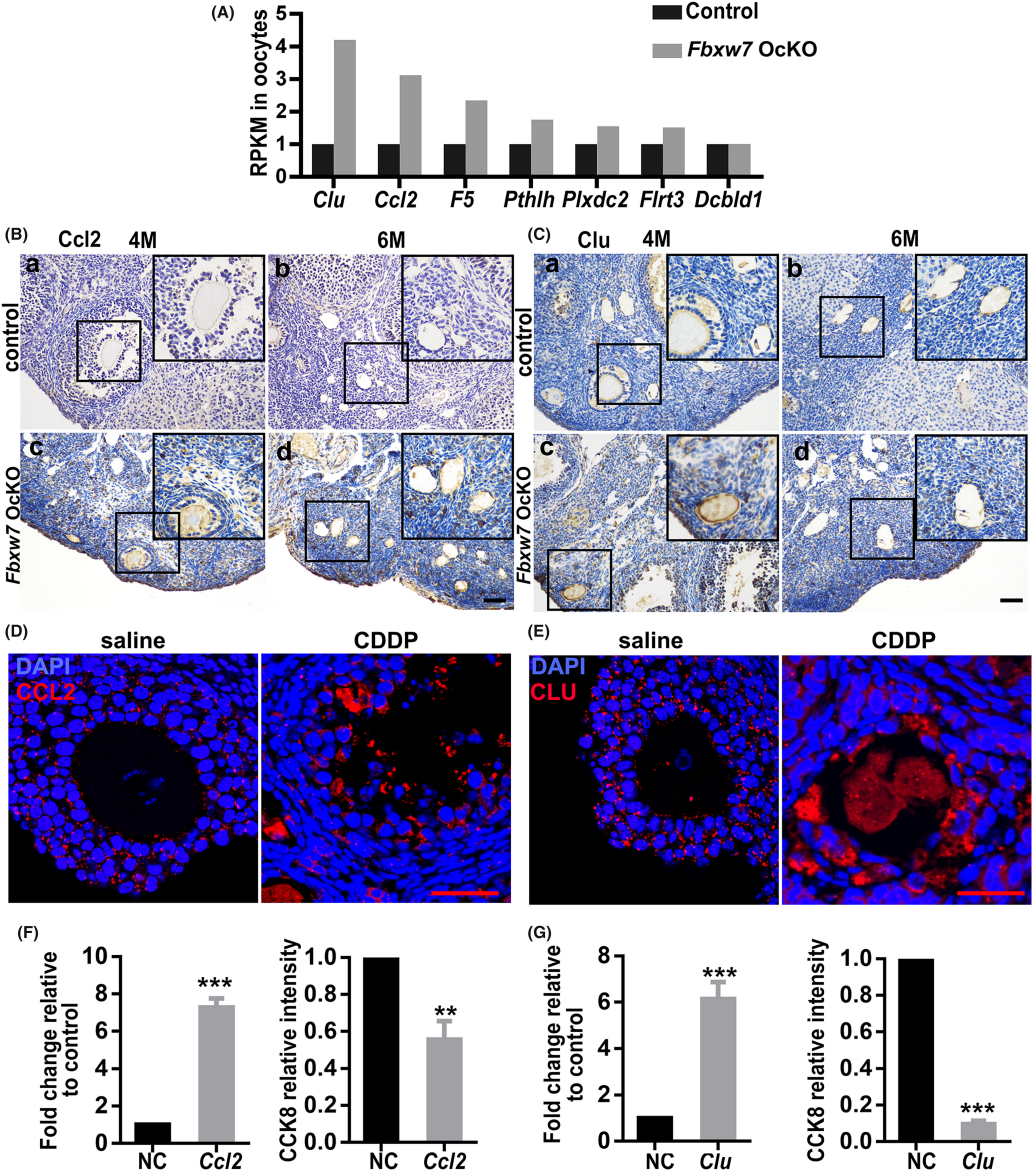
Deletion of *Fbxw7* causes aberrant elevation of the transcripts of *Ccl2* and *Clu*. (A) Alteration of the target genes via single‐cell RNA‐sequencing analysis. (B, C) Expression of *Ccl2* and *Clu* in the ovaries of paired mice by in situ hybridization. (D, E) CCL2 and CLU expression patterns in follicles of saline‐ and CDDP‐treated POI mice. (F, G) CCL2 and CLU overexpressed KGN cells viability determined by CCK8. Data were presented as mean values ± SEM. *n* = 5 for each group, scale bar = 50 μm. *, *p* < 0.05; **, *p* < 0.01, ***, *p* < 0.001, compared with control.

To explore the FBXW7 pathways in oocyte maturation process, we performed single‐cell RNA sequencing (RNA‐seq) with 137 *Fbxw7*‐depleted MII‐oocytes and 134 wild‐type MII‐oocytes (Figure 6A). The results indicate a significant upregulation of *Clu* and *Ccl2* in the *Fbxw7*‐depleted oocytes, with fold increases of 4.2 and 3.1, respectively.

By conducting in situ hybridization experiments, we observed an aberrant upregulation of *Ccl2* and *Clu* expressions in both oocytes and GCs within *Fbxw7* OcKO ovaries (Figure 6B,C). Immunofluorescence showed that following CDDP treatment, both CCL2 and CLU were also aberrantly upregulated in both oocytes and GCs, indicating a mechanistic consistency between CDDP‐induced and *Fbxw7*‐depletion‐induced POI (Figure 6D,E). To assess the effects of CCL2 and CLU elevation on human GCs, we utilized the human GC line KGN, overexpressed CCL2 and CLU separately, and measured cell viability (Figure 6F,G). The results showed that both overexpression of CCL2 and CLU significantly compromised cell viability. Therefore, loss of *Fbxw7* in oocytes leads to follicular damage by upregulating *Ccl2* and *Clu* in both oocytes and GCs.

## 4 DISCUSSION

Cisplatin (CDDP) is a commonly used chemotherapy drug for treating various types of tumours, including bladder cancer, cervical cancer, breast cancer, and ovarian cancer.^27^, ^28^ While chemotherapy has improved the long‐term survival rates of young cancer patients, it has also increased the risk of early‐onset ovarian dysfunction, known as Primary Ovarian Insufficiency (POI).^29^ Ovarian function loss resulting from POI has significant implications for quality of life, bone health, as well as cardiovascular and neurological system health. POI stands as a significant contributor to reduced female fertility.^19^, ^30^, ^31^ CDDP is also a commonly used modelling method for the study of POI, and has been widely reported and used. Existing research has shown promising rescue effects of hormones, vitamin E, stem cells, micro‐RNAs, and extracellular vesicles on POI^30^, ^31^, ^32^, ^33^, ^34^ However, these interventions primarily target ovarian stromal cells and granulosa cells (GCs) within the follicles.^35^ The mechanisms underlying POI remain unclear, and effective predictive and therapeutic strategies are lacking. Therefore, investigating the mechanisms of CDDP damage and diagnosing the extent of POI is paramount importance. Researchers have indicated that inhibiting CK1 activity mitigates CDDP‐induced damage to primary oocytes.^35^, ^36^ Here, we observed reduced FBXW7 expression in oocytes following CDDP treatment. Thus, we hypothesized that downregulation of FBXW7 in oocytes plays a role in the development of POI.

FBXW7 comprises three isoforms, namely, FBXW7 α, −β, and ‐γ, all sharing the WD40 repeat structure. These isoforms exhibit distinct subcellular localizations: FBXW7 α in the nucleus, FBXW7 β in the cytoplasm, and FBXW7 γ in the nucleolus.^20^ Our findings indicate that FBXW7 primarily exists as α and β isoforms in oocytes. Research has demonstrated that deletion of FBXW7 in tumour cells leads to mitotic defects and chromosomal instability, promoting tumorigenesis.^37^ Our previous work established the role of FBXW7 in Sertoli cell differentiation in male gonads, with its absence disturbs Sertoli cell differentiation and thus disrupts testicular development.^20^ Similarly, in the nematode Caenorhabditis elegans, the FBXW7 homologue SEL‐10 coordinates meiosis and post‐transcriptional gene expression during gametogenesis through the MPK‐1/MAPK pathway.^38^ Moreover, during primordial follicle development, FBXW7 inhibits primordial follicle activation via the eNOS/cGMP/PKG pathway.^39^ These researches demonstrate the vital regulatory role of FBXW7 in cell cycle, particularly in the germ cell cycle, which corroborates our findings. For the first time, our experiments demonstrated the association between normal FBXW7 function with folliculogenesis and ovarian function, and its dysfunction is largely implicated in POI, thus reinforcing our initial hypothesis.

Further investigations reveal alterations in gap junctions between oocytes and GCs, while gap junctions among GCs themselves remain largely unchanged. Immunofluorescence results indicate no significant reduction in FBXW7 expression in GCs. This suggests that the occurrence of POI is not directly linked to decreased FBXW7 expression in GCs. Therefore, we reasoned that oocytes regulate the functions of GCs through downstream factors shared with GCs, which is probably modulated by FBXW7.

Therefore, we performed single‐cell RNA‐seq combined with in situ hybridization analysis of MII stage oocytes to investigate the mechanism of CDDP‐induced oocyte damage. Growing oocytes exhibit transcriptional activity, which becomes inactive once their growth phase ends (fully grown GV), allowing them to complete their meiotic division process (MII stage). During the transition from GV to MII, specific transcripts associated with the MII maturation pathway are protected from degradation.^40^ Thus, the transition from GV to MII involves the depletion of specific transcripts associated with oocyte maturation pathways, and the aberrant expression that may cause oocyte damage could be identified by screening MII stage oocytes. Our RNA‐seq results indicate that the highly expressed mRNAs during the MII stage (*Clu* and *Ccl2*) are likely specific transcripts responsible for POI damage induced by *Fbxw7* deficiency. To confirm that CCL2 and CLU are not only differentially expressed in the MII stage, but also involved in oocyte damage. We performed in situ hybridization analysis on mature healthy follicles. The results suggest that CCL2 and CLU likely play a role throughout the entire follicular development period, because elevated expression of CLU and CCL2 were observed in both oocytes and granulosa cells within *Fbxw7* OcKO ovaries.

We emphasize the role of CLU and CCL2, which show specific upregulation in MII stage oocytes of *Fbxw7* OcKO. Previous studies have highlighted Clusterin as a highly sulfated heterodimeric glycoprotein expressed in the human ovary and bodily fluids.^41^, ^42^ CLU has been mentioned as a novel biomarker in ovarian cancer research.^43^ Studies have shown that intraperitoneal injection of PMSG induces follicular arrest, and *Clu* is highly expressed in apoptotic atresia follicles.^44^ CCL2, a member of monocyte chemotactic proteins, contains two closely linked 2‐Cys in its N‐terminus.^45^ CCL2 exhibits high expression in GCs of patients with polycystic ovary syndrome (PCOS).^46^, ^47^ Furthermore, CCL2 serves as a crucial marker for ovarian angiogenesis and development.^48^ This further suggests the involvement of CLU and CCL2 in abnormal follicular development. Our study suggests that the upregulation of CLU and CCL2 in GCs are caused by abnormal oocytes. Additionally, we verified that overexpression of *Clu* and *Ccl2* in human ovarian GCs leads to increased GC apoptosis (Figure 6E,G). Our study reveals that abnormal oocyte development can directly induce GCs apoptosis through paracrine signalling, finally leading to POI.

However, whether FBXW7 directly targets CCL2 and CLU to mediate ovarian dysfunction is still unclear. While our study provides compelling evidence supporting the role of FBXW7 in regulating GC and follicular damage in oocytes, Therefore, in the discussion, we emphasize the need for further research in this area. Based on the current understanding of FBXW7, it is a member of the ubiquitin ligase E3 family,^49^ promoting substrate protein degradation by ubiquitination and promote their degradation through the proteasome system. However, the observed a high expression of CCL2 and CLU simultaneously, that seems inconsistent with the function of FBXW7. Therefore, we speculate that FBXW7 might regulate a shared upstream protein of CCL2 and CLU, causing their upregulation. We screened MCM2 as a potential interacting protein of FBXW7, CCL2, and CLU through the Biogrid protein interaction database, but this relationship remains speculative. MCM2 is a subunit of the minichromosome maintenance (MCM) complex, crucial for DNA replication initiation and maintaining genomic stability.^50^ It has been shown that irisin induces simultaneous upregulation of MCM2 and CCL2 in C2C12 myoblasts.^51^ This agrees with our speculation that FBXW7 may ubiquitinate and degrade MCM2 to regulate CCL2 and CLU expression. Deletion of *Fbxw7* may cause accumulation of MCM2, resulting in increased CCL2 and CLU expression.

In summary, our research underscores the critical role of FBXW7 in oocytes for follicular development, with its loss leading to POI. Further exploration of the mechanisms underlying FBXW7 functions and its associations with CCL2 and CLU are expected to understand the pathogenesis and diagnosis of POI, providing new perspectives for the prevention and treatment of POI.

## AUTHOR CONTRIBUTIONS

**Huihui Zhao:** Conceptualization (equal); data curation (equal); formal analysis (equal); investigation (equal); methodology (equal); project administration (equal); resources (equal); software (equal); supervision (equal); validation (equal); visualization (equal); writing – original draft (equal); writing – review and editing (equal). **Hanbin Zhang:** Data curation (equal); investigation (equal); methodology (equal); project administration (equal); resources (equal); software (equal); supervision (equal); writing – original draft (equal); writing – review and editing (equal). **Yuxia Zhou:** Data curation (equal); methodology (equal); software (equal); writing – original draft (equal). **Ling Shuai:** Methodology (equal); project administration (equal); software (equal). **Zhenguo Chen:** Conceptualization (equal); formal analysis (equal); funding acquisition (equal); investigation (equal); project administration (equal); supervision (equal); writing – review and editing (equal). **Liping Wang:** Conceptualization (equal); formal analysis (equal); funding acquisition (equal); investigation (equal); supervision (equal); writing – review and editing (equal).

## FUNDING INFORMATION

The study was supported by the National Natural Science Foundation of China (grant number: 81571389, 81401258), Guangdong Basic and Applied Basic Research Foundation (grant number: 2019A1515011693) and Medical Scientific Research Foundation of Guangdong (grant number: A2019337).

## CONFLICT OF INTEREST STATEMENT

The authors declared no conflict of interests.

## Data Availability

Data openly available in a public repository that issues datasets with DOIs.

## References

[1] Ge W , Li L , Dyce PW , De Felici M , Shen W . Establishment and depletion of the ovarian reserve: physiology and impact of environmental chemicals. Cell Mol Life Sci. 2019;76(9):1729‐1746. doi:10.1007/s00018-019-03028-1 30810760 PMC11105173

[2] European Society for Human R, Embryology Guideline Group on POI , Webber L , et al. ESHRE guideline: management of women with premature ovarian insufficiency. Hum Reprod. 2016;31(5):926‐937. doi:10.1093/humrep/dew027 27008889

[3] Yang X , Zhang X , Jiao J , et al. Rare variants in FANCA induce premature ovarian insufficiency. Hum Genet. 2019;138(11–12):1227‐1236. doi:10.1007/s00439-019-02059-9 31535215 PMC6874525

[4] Hernandez‐Angeles C , Castelo‐Branco C . Early menopause: a hazard to a woman's health. Indian J Med Res. 2016;143(4):420‐427. doi:10.4103/0971-5916.184283 27377497 PMC4928547

[5] Chu K , He Y , Li Z , et al. Novel LAT pathogenic variants in a POI family and its role in the ovary. Front Genet. 2021;12:764160. doi:10.3389/fgene.2021.764160 34868246 PMC8640088

[6] Qin Y , Jiao X , Simpson JL , Chen ZJ . Genetics of primary ovarian insufficiency: new developments and opportunities. Hum Reprod. 2015;21(6):787‐808. doi:10.1093/humupd/dmv036 PMC4594617 26243799

[7] Meczekalski B , Podfigurna‐Stopa A . Genetics of premature ovarian failure. Minerva Endocrinol. 2010;35(4):195‐209. 21178916

[8] Yeh CH , Bellon M , Nicot C . FBXW7: a critical tumor suppressor of human cancers. Mol Cancer. 2018;17(1):115. doi:10.1186/s12943-018-0857-2 30086763 PMC6081812

[9] Sailo BL , Banik K , Girisa S , et al. FBXW7 in cancer: what has been unraveled thus far? Cancer. 2019;11(2):246. doi:10.3390/cancers11020246 PMC6406609 30791487

[10] Mao JH , Perez‐Losada J , Wu D , et al. Fbxw7/Cdc4 is a p53‐dependent, haploinsufficient tumour suppressor gene. Nature. 2004;432(7018):775‐779. doi:10.1038/nature03155 15592418

[11] Kanatsu‐Shinohara M , Onoyama I , Nakayama KI , Shinohara T . Skp1‐Cullin‐F‐box (SCF)‐type ubiquitin ligase FBXW7 negatively regulates spermatogonial stem cell self‐renewal. Proc Natl Acad Sci USA. 2014;111(24):8826‐8831. doi:10.1073/pnas.1401837111 24879440 PMC4066470

[12] Hsueh AJ , Kawamura K , Cheng Y , Fauser BC . Intraovarian control of early folliculogenesis. Endocr Rev. 2015;36(1):1‐24. doi:10.1210/er.2014-1020 25202833 PMC4309737

[13] Ford EA , Beckett EL , Roman SD , McLaughlin EA , Sutherland JM . Advances in human primordial follicle activation and premature ovarian insufficiency. Reproduction. 2020;159(1):R15‐R29. doi:10.1530/REP-19-0201 31376814

[14] Atwood CS , Vadakkadath MS . The spatiotemporal hormonal orchestration of human folliculogenesis, early embryogenesis and blastocyst implantation. Mol Cell Endocrinol. 2016;430:33‐48. doi:10.1016/j.mce.2016.03.039 27045358

[15] Baba T , Ting AY , Tkachenko O , Xu J , Stouffer RL . Direct actions of androgen, estrogen and anti‐Mullerian hormone on primate secondary follicle development in the absence of FSH in vitro. Hum Reprod. 2017;32(12):2456‐2464. doi:10.1093/humrep/dex322 29077845 PMC6075619

[16] Gershon E , Plaks V , Dekel N . Gap junctions in the ovary: expression, localization and function. Mol Cell Endocrinol. 2008;282(1–2):18‐25. doi:10.1016/j.mce.2007.11.001 18162286

[17] Li SY , Bhandary B , Gu X , DeFalco T . Perivascular cells support folliculogenesis in the developing ovary. Proc Natl Acad Sci USA. 2022;119(41):e2213026119. doi:10.1073/pnas.2213026119 36194632 PMC9564831

[18] Wang H , Cai H , Wang X , et al. HDAC3 maintains oocyte meiosis arrest by repressing amphiregulin expression before the LH surge. Nat Commun. 2019;10(1):5719. doi:10.1038/s41467-019-13671-8 31844300 PMC6915726

[19] Wu Y , Ma C , Zhao H , Zhou Y , Chen Z , Wang L . Alleviation of endoplasmic reticulum stress protects against cisplatin‐induced ovarian damage. Reprod Biol Endocrinol. 2018;16(1):85. doi:10.1186/s12958-018-0404-4 30176887 PMC6122480

[20] Zhang H , Chen F , Dong H , et al. Loss of Fbxw7 in Sertoli cells impairs testis development and causes infertility in mice†. Biol Reprod. 2020;102(4):963‐974. doi:10.1093/biolre/ioz230 31883011

[21] Chang EM , Lim E , Yoon S , et al. Cisplatin induces overactivation of the dormant primordial follicle through PTEN/AKT/FOXO3a pathway which leads to loss of ovarian Reserve in Mice. PLoS One. 2015;10(12):e0144245. doi:10.1371/journal.pone.0144245 26656301 PMC4699462

[22] Liu XM , Yan MQ , Ji SY , et al. Loss of oocyte Rps26 in mice arrests oocyte growth and causes premature ovarian failure. Cell Death Dis. 2018;9(12):1144. doi:10.1038/s41419-018-1196-3 30451825 PMC6242890

[23] Liu Y , Jiang Q , Liu X , et al. Cinobufotalin powerfully reversed EBV‐miR‐BART22‐induced cisplatin resistance via stimulating MAP2K4 to antagonize non‐muscle myosin heavy chain IIA/glycogen synthase 3beta/beta‐catenin signaling pathway. EBioMedicine. 2019;48:386‐404. doi:10.1016/j.ebiom.2019.08.040 31594754 PMC6838365

[24] Ten J , Mendiola J , Vioque J , de Juan J , Bernabeu R . Donor oocyte dysmorphisms and their influence on fertilization and embryo quality. Reprod Biomed Online. 2007;14(1):40‐48. doi:10.1016/s1472-6483(10)60762-6 17207330

[25] Bassil R , Casper RF , Meriano J , et al. Can oocyte diameter predict embryo quality? Reprod Sci. 2021;28(3):904‐908. doi:10.1007/s43032-020-00306-3 32876908

[26] Timlin CL , Lynn A , Wooldridge LK , et al. Physical parameters of bovine activated oocytes and zygotes as predictors of development success. Zygote. 2021;29(5):358‐364. doi:10.1017/S0967199421000058 33736736

[27] Loren P , Saavedra N , Saavedra K , et al. Contribution of MicroRNAs in chemoresistance to cisplatin in the top five deadliest cancer: an updated review. Front Pharmacol. 2022;13:831099. doi:10.3389/fphar.2022.831099 35444536 PMC9015654

[28] Ge Y , Zheng N , Chen X , et al. GMDTC chelating agent attenuates cisplatin‐induced systemic toxicity without affecting antitumor efficacy. Chem Res Toxicol. 2019;32(8):1572‐1582. doi:10.1021/acs.chemrestox.9b00097 31240907

[29] Meirow D . Reproduction post‐chemotherapy in young cancer patients. Mol Cell Endocrinol. 2000;169(1–2):123‐131. doi:10.1016/s0303-7207(00)00365-8 11155944

[30] Zhao H , Gu W , Pan W , et al. miR‐483‐5p aggravates cisplatin‐induced premature ovarian insufficiency in rats by targeting FKBP4. Nan Fang Yi Ke Da Xue Xue Bao. 2021;41(6):801‐810. doi:10.12122/j.issn.1673-4254.2021.06.01 34238731 PMC8267993

[31] Sun L , Li D , Song K , et al. Exosomes derived from human umbilical cord mesenchymal stem cells protect against cisplatin‐induced ovarian granulosa cell stress and apoptosis in vitro. Sci Rep. 2017;7(1):2552. doi:10.1038/s41598-017-02786-x 28566720 PMC5451424

[32] Del Castillo LM , Buigues A , Rossi V , et al. The cyto‐protective effects of LH on ovarian reserve and female fertility during exposure to gonadotoxic alkylating agents in an adult mouse model. Hum Reprod. 2021;36(9):2514‐2528. doi:10.1093/humrep/deab165 34333622 PMC8373474

[33] Du R , Cheng X , Ji J , et al. Mechanism of ferroptosis in a rat model of premature ovarian insufficiency induced by cisplatin. Sci Rep. 2023;13(1):4463. doi:10.1038/s41598-023-31712-7 36932163 PMC10023701

[34] Lu X , Bao H , Cui L , et al. hUMSC transplantation restores ovarian function in POI rats by inhibiting autophagy of theca‐interstitial cells via the AMPK/mTOR signaling pathway. Stem Cell Res Ther. 2020;11(1):268. doi:10.1186/s13287-020-01784-7 32620136 PMC7333437

[35] Tuppi M , Kehrloesser S , Coutandin DW , et al. Oocyte DNA damage quality control requires consecutive interplay of CHK2 and CK1 to activate p63. Nat Struct Mol Biol. 2018;25(3):261‐269. doi:10.1038/s41594-018-0035-7 29483652

[36] Kim SY , Nair DM , Romero M , et al. Transient inhibition of p53 homologs protects ovarian function from two distinct apoptotic pathways triggered by anticancer therapies. Cell Death Differ. 2019;26(3):502‐515. doi:10.1038/s41418-018-0151-2 29988075 PMC6370889

[37] Takada M , Zhang W , Suzuki A , et al. FBW7 loss promotes chromosomal instability and tumorigenesis via cyclin E1/CDK2‐mediated phosphorylation of CENP‐A. Cancer Res. 2017;77(18):4881‐4893. doi:10.1158/0008-5472.CAN-17-1240 28760857 PMC5743019

[38] Kisielnicka E , Minasaki R , Eckmann CR . MAPK signaling couples SCF‐mediated degradation of translational regulators to oocyte meiotic progression. Proc Natl Acad Sci USA. 2018;115(12):E2772‐E2781. doi:10.1073/pnas.1715439115 29496961 PMC5866554

[39] Zhao P , Song Z , Wang Y , et al. The endothelial nitric oxide synthase/cyclic guanosine monophosphate/protein kinase G pathway activates primordial follicles. Aging (Albany NY). 2020;13(1):1096‐1119. doi:10.18632/aging.202235 33291075 PMC7835019

[40] Llonch S , Barragan M , Nieto P , et al. Single human oocyte transcriptome analysis reveals distinct maturation stage‐dependent pathways impacted by age. Aging Cell. 2021;20(5):e13360. doi:10.1111/acel.13360 33908703 PMC8135014

[41] Jarkovska K , Martinkova J , Liskova L , et al. Proteome mining of human follicular fluid reveals a crucial role of complement cascade and key biological pathways in women undergoing in vitro fertilization. J Proteome Res. 2010;9(3):1289‐1301. doi:10.1021/pr900802u 20058866

[42] Kovacs P , Pushparaj PN , Takacs R , Mobasheri A , Matta C . The clusterin connectome: emerging players in chondrocyte biology and putative exploratory biomarkers of osteoarthritis. Front Immunol. 2023;14:1103097. doi:10.3389/fimmu.2023.1103097 37033956 PMC10081159

[43] Gunawardana CG , Kuk C , Smith CR , Batruch I , Soosaipillai A , Diamandis EP . Comprehensive analysis of conditioned media from ovarian cancer cell lines identifies novel candidate markers of epithelial ovarian cancer. J Proteome Res. 2009;8(10):4705‐4713. doi:10.1021/pr900411g 19663500

[44] Zwain I , Amato P . Clusterin protects granulosa cells from apoptotic cell death during follicular atresia. Exp Cell Res. 2000;257(1):101‐110. doi:10.1006/excr.2000.4885 10854058

[45] Zahner G , Schaper M , Panzer U , et al. Prostaglandin EP2 and EP4 receptors modulate expression of the chemokine CCL2 (MCP‐1) in response to LPS‐induced renal glomerular inflammation. Biochem J. 2009;422(3):563‐570. doi:10.1042/BJ20090420 19570035

[46] Schmidt J , Weijdegard B , Mikkelsen AL , Lindenberg S , Nilsson L , Brannstrom M . Differential expression of inflammation‐related genes in the ovarian stroma and granulosa cells of PCOS women. Mol Hum Reprod. 2014;20(1):49‐58. doi:10.1093/molehr/gat051 23900753

[47] Chen H , Cheng S , Xiong W , Tan X . The lncRNA‐miRNA‐mRNA ceRNA network in mural granulosa cells of patients with polycystic ovary syndrome: an analysis of gene expression omnibus data. Ann Transl Med. 2021;9(14):1156. doi:10.21037/atm-21-2696 34430597 PMC8350636

[48] Chermula B , Brazert M , Izycki D , et al. New gene markers of angiogenesis and blood vessels development in porcine ovarian granulosa cells during short‐term primary culture in vitro. Biomed Res Int. 2019;2019:6545210. doi:10.1155/2019/6545210 30834271 PMC6374792

[49] Hao B , Oehlmann S , Sowa ME , Harper JW , Pavletich NP . Structure of a Fbw7‐Skp1‐cyclin E complex: multisite‐phosphorylated substrate recognition by SCF ubiquitin ligases. Mol Cell. 2007;26(1):131‐143. doi:10.1016/j.molcel.2007.02.022 17434132

[50] Cvetkovic MA , Passaretti P , Butryn A , et al. The structural mechanism of dimeric DONSON in replicative helicase activation. Mol Cell. 2023;83(22):4017‐4031. doi:10.1016/j.molcel.2023.09.029 37820732 PMC7616792

[51] Lee J , Park J , Kim YH , Lee NH , Song KM . Irisin promotes C2C12 myoblast proliferation via ERK‐dependent CCL7 upregulation. PLoS One. 2019;14(9):e0222559. doi:10.1371/journal.pone.0222559 31518371 PMC6743866

